# Pprevalence and antibiotic resistance profile of bacterial isolates from wounds in burn unit and a nearby trauma ward at Ramathibodi Hopsital, Bangkok, Thailand

**DOI:** 10.1186/1753-6561-5-S6-P230

**Published:** 2011-06-29

**Authors:** S Srisangkaew, A Boonnark, M Kunakorn, P Santanirand

**Affiliations:** 1 Department of pathology, Ramathibodi Hospital, Mahidol University, Bangkok, Thailand

## Introduction / objectives

Data analysis of prevalence and antibiotic resistance profile of bacterial isolates from the particular wards could lead to an improvement of treatment, control and prevention of infection in the units. The aim of this study is to evaluate predominant bacterial isolates from the Burn Unit (BU) and a nearby trauma ward (TW) in Ramathibodi hospital, Bangkok, Thailand.

## Methods

A five-year retrospective review of wound cultures from patients who admitted at BU and a nearby TW, Ramathibodi hospital was performed.

## Results

The highest prevalence Gram-negative and Gram-positive bacteria in both wards were similar as *Pseudomonas aeruginosa* (20.99 and 15.17%) and *Staphylococcus aureus* (13.12 and 12.16%), respectively. Although the prevalence of multiple drug resistant (MDR) strains of *Acinetobacter baumannii* showed no differrence (59.09 vs 60.53%) in both wards, the prevelence of MDR *P. aeruginosa* and methicillin-resistant *S. aureus* (MRSA) in BU was higher than TW (15.28 vs 6.25% and 66.67 vs 51.95%, respectively). In contrast, the prevalence of *E. coli* and *Enterobacter* spp. isolated from TW was higher than BU whereas the second common Gram-positive organism was *Enterococcus* spp. in both wards.

## Conclusion

Not only high incidence of bacterial infection but also high prevalence of MDR strains in Burn Unit has become another challenge for medical staff as well as those who involve in hospital infection control program to be seriously concerned.

## Disclosure of interest

None declared.

